# Intrinsic Antibacterial Urushiol-Based Benzoxazine Polymer Coating for Marine Antifouling Applications

**DOI:** 10.3390/ijms26094118

**Published:** 2025-04-26

**Authors:** Nuo Chen, Jide Zhu, Xinrong Chen, Fengcai Lin, Xiaoxiao Zheng, Guocai Zheng, Qi Lin, Jipeng Chen, Yanlian Xu

**Affiliations:** 1 Fujian Engineering Research Center of New Chinese Lacquer Materials, College of Materials and Chemical Engineering, Minjiang University, Fuzhou 350108, China; ccccheng7788@outlook.com (N.C.); 13599930822@163.com (X.C.); fengcailin@mju.edu.cn (F.L.); xxzheng@mju.edu.cn (X.Z.); 2231@mju.edu.cn (G.Z.); qlin1990@163.com (Q.L.); 2 National Engineering Research Center of Chemical Fertilizer Catalyst (NERC-CFC), College of Chemical Engineering, Fuzhou University, Fuzhou 350002, China; zjd320610217@163.com

**Keywords:** urushiol-based benzoxazine polymer, rosin amine, intrinsic antibacterial, marine antifouling, ring-opening polymerization

## Abstract

Marine antifouling coatings that rely on the release of antifouling agents are the most prevalent and effective strategy for combating fouling. However, the environmental concerns arising from the widespread discharge of these agents into marine ecosystems cannot be overlooked. An innovative and promising alternative involves incorporating antimicrobial groups into polymers to create coatings endowed with intrinsic antimicrobial properties. In this study, we reported an urushiol-based benzoxazine (URB) monomer, synthesized from natural urushiol and antibacterial rosin amine. The URB monomer was subsequently polymerized through thermal curing ring-opening polymerization, resulting in the formation of a urushiol-based benzoxazine polymer (URHP) coating with inherent antimicrobial properties. The surface of the URHP coating is smooth, flat, and non-permeable. Contact angle and surface energy measurements confirm that the URHP coating is hydrophobic with low surface energy. In the absence of antimicrobial agent release, the intrinsic properties of the URHP coating can effectively kill or repel fouling organisms. Furthermore, with bare glass slides serving as the control sample, the coating demonstrates outstanding anti-adhesion capabilities against four types of bacteria (*E. coli*, *S. aureus*, *V. alginolyticus*, and *Bacillus* sp.), and three marine microalgae (*N. closterium*, *P. tricornutum*, and *D. zhan-jiangensis*), proving its efficacy in preventing fouling organisms from settling and adhering to the surface. Thus, the combined antibacterial and anti-adhesion properties endow the URHP coating with superior antifouling performance. This non-release antifouling coating represents a green and environmentally sustainable strategy for antifouling.

## 1. Introduction

The damage and losses inflicted by marine biofouling are considerable, prompting substantial investments in the development of marine antifouling technologies [1,2]. Among the various strategies employed to combat marine biofouling, the application of antifouling coatings stands out as the most straightforward, cost-effective, and efficient approach [3]. The two most prevalent antifouling mechanisms of these coatings are as follows: (i) the release of antifouling agents that effectively kill or repel fouling organisms; and (ii) the modification of the physicochemical properties of the coating to impart unique surface characteristics, thereby creating a “barrier” on the surface that prevents the adhesion of fouling organisms or facilitates their easy detachment after adhesion, thus achieving the desired antifouling effect [4,5,6].

At present, numerous antifouling resins with distinct physicochemical properties have been developed, including acrylic self-polishing resins, degradable polymers, biomimetic antifouling materials, anti-protein adhesion hydrophilic materials, and low surface energy antifouling materials [1,7,8,9]. Different antifouling resin systems exhibit their own unique characteristics and advantages yet also present certain limitations. For example, regulating the degradation rate and ensuring the prolonged efficacy of degradable polymer antifouling coatings presents a significant challenge. The fabrication of expansive surfaces with distinctive biomimetic properties remains a formidable task for biomimetic materials. Anti-protein adhesion hydrophilic antifouling coatings often exhibit subpar mechanical performance. Meanwhile, the low surface energy leads to less-than-ideal static antifouling effectiveness and complications during the coating processes. Acrylic self-polishing resins, furthermore, are the most widely employed antifouling resins in commercial coatings. These resins rely on the hydrolysis of ester groups within their structure, causing the polymer matrix to gradually dissolve and detach, along with the fouling organisms, thus facilitating surface renewal. This results in effective dynamic antifouling performance. However, the utilization of antifouling agents is substantial (with Cu_2_O content typically exceeding 40%) [10,11], and the long-term effectiveness remains challenging to regulate. On the other hand, hydrolysis products have been identified as one of the primary sources of microplastics in the ocean, and the detrimental effects of microplastics have been extensively documented [1,12]. Furthermore, the widespread utilization of antifouling agents such as copper oxide and copper pyrithione, which are inherently toxic and harmful substances, poses a significant threat to non-target organisms, resulting in severe damage to the ecological environment [13]. Consequently, with the growing emphasis on environmental conservation, the use of release-type marine antifouling coatings is expected to face increasing restrictions, while the development of eco-friendly, non-release-type marine antifouling coatings has emerged as a focal point of research.

By integrating bio-sensitive antimicrobial groups into the structure of antifouling resins or grafting antimicrobial antifouling agents onto the surface of coatings, such as quaternary ammonium salts [14,15,16], triclosan [17,18], isothiazolinone [19,20], and N-(2,4,6-trichlorophenyl) maleimide [5,21,22], the antifouling action can be seamlessly incorporated into the resin or coating. This approach imparts inherent antimicrobial properties to the coating, allowing it to eliminate or deter fouling organisms without the need for the release of antimicrobial substances or antifouling agents, thereby achieving effective antifouling. Moreover, it typically influences only the fouling organisms in direct proximity to or in contact with the coating surface, without affecting marine life in other regions, thereby presenting a sustainable, eco-friendly, and long-lasting antifouling solution. This coating preserves the original physicochemical properties of the polymer resin while augmenting its antimicrobial capabilities, resulting in a coating with robust dynamic antifouling performance, which is further enhanced by static antifouling properties. As a result, the coating achieves both dynamic and static antifouling effectiveness. However, coatings that incorporate antifouling agents via grafting are predominantly derived from petroleum-based materials, and the grafting process, typically involving multiple intricate steps on the resin surface, results in increased energy consumption, thereby limiting their application.

**Figure 1:**
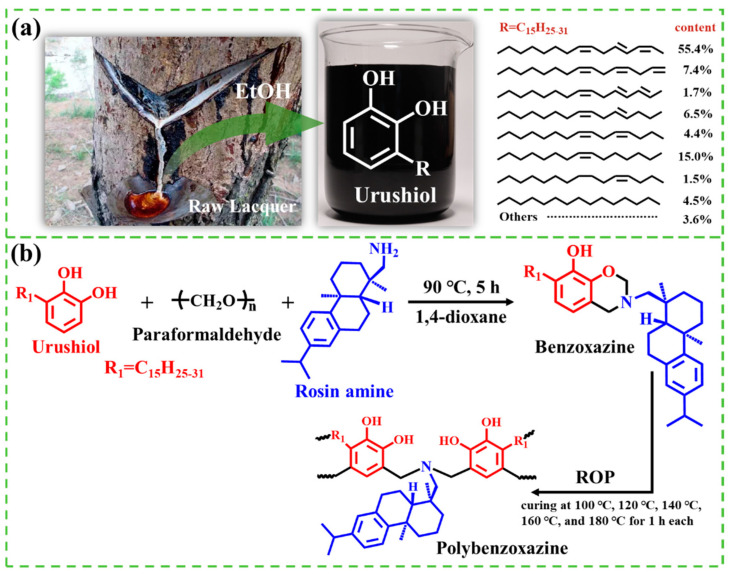
(**a**) Collection of raw lacquer and chemical structure of urushiol; (**b**) synthetic route of urushiol–rosin amine–benzoxazine monomer and polymer.

In contrast to petroleum-based materials, bio-based materials are increasingly preferred by researchers for their renewable and environmentally sustainable attributes, making them a focal point of research in recent years [23,24,25]. Urushiol, the principal component of the secretion from the lacquer tree, commonly referred to as raw lacquer (Figure 1a), is a natural, eco-friendly bio-based substance. Its structure, resembling that of dopamine, comprises an unsaturated long carbon chain and a catechol structure, showcasing a range of exceptional properties [26,27]. Our previous studies [28,29] detailed the synthesis of low-surface-energy urushiol-based benzoxazine polymers with varied structures, utilizing urushiol as the primary raw material, and their subsequent application in marine antifouling coatings. Experimental findings revealed that the low-surface-energy urushiol-based benzoxazine polymer coatings exhibited exceptional antifouling performance. Additionally, capitalized on the flexible molecular design capabilities of benzoxazine, bioactive components can be integrated into the polymer backbone during the synthesis of benzoxazine resins, thus eliminating the need for cumbersome post-modification processes and offering a straightforward and efficient preparation method.

Building upon this, leveraging the flexible molecular design capabilities of benzoxazine, we further employed urushiol and antibacterial rosin amine as raw materials to synthesize a urushiol-based benzoxazine polymer coating endowed with intrinsic antimicrobial properties. The structure and performance of the urushiol-based benzoxazine polymer coating were thoroughly characterized. The antifouling efficacy of the coating was assessed using a range of typical fouling organisms, including various bacteria and marine microalgae. In the absence of antimicrobial agent release, the intrinsic antimicrobial properties of the coating were harnessed to eradicate or repel fouling organisms, thereby preventing their settlement and adhesion on the surface, and ultimately enhancing the static antifouling performance of the benzoxazine polymer coating. This study thus provides an experimental foundation for the development and design of non-leaching, environmentally friendly, and efficient marine antifouling coatings.

## 2. Results and Discussion

### 2.1. Synthesis and Characterization of URB Monomer

Leveraging the flexible molecular design of benzoxazine, functional groups are introduced into its structure to functionalize the coating and endow it with unique properties. Consequently, rosin amine (RA), known for its antimicrobial and anti-algal effects, was employed as the amine source and reacted with urushiol (U) and paraformaldehyde to synthesize a novel bio-based benzoxazine monomer (URB). This monomer was subsequently polymerized through thermosetting ring-opening polymerization (ROP) to yield a urushiol–rosin amine–benzoxazine polymer (URHP) coating, endowed with intrinsic antimicrobial properties [29,30,31], as shown in Figure 1b.

**Figure 2:**
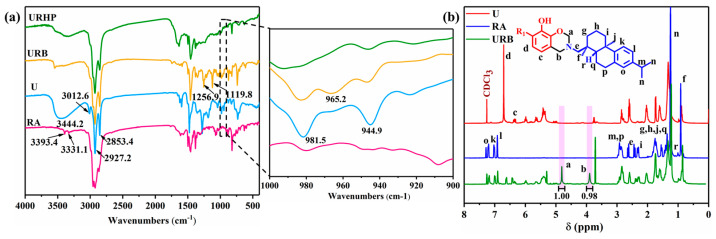
(**a**) ATR-FTIR spectra of U, RA, URB, and URHP; (**b**) ^1^H NMR spectra of U, RA, and URB monomer; 1.00 and 0.98 represent the integration of peaks a and b.

The ATR-FTIR spectra of U and URB are presented in Figure 2a. The broad absorption band observed in the range of 3300–3400 cm^−1^ corresponds to the phenolic hydroxyl group (–OH), while the characteristic absorption bands at 3012.6, 2927.2, and 2853.4 cm^−1^ represent the stretching vibrations of isolated unsaturated =C–H groups and the stretching vibrations of saturated alkyl C–H groups, respectively. Furthermore, the absorption peak at 1651.5 cm^−1^ is attributed to the stretching vibrations of C=O, while the peaks at 1591.8 and 1455.5 cm^−1^ correspond to the C=C stretching vibrations. The characteristic absorption peaks at 1256.9 cm^−1^ and 1119.8 cm^−1^ are ascribed to the asymmetric stretching vibrations of the Ar–O–C bond in the oxazine ring and the asymmetric stretching vibrations of the C–N–C bond in the benzoxazine structure, respectively. Moreover, in the partial enlargement of the ATR-FTIR spectrum, the characteristic peaks associated with the oxazine ring structure of URB are observed at 965.2 cm^−1^. The distinct absorption peaks at 981.5 and 944.9 cm^−1^ are attributed to the in-plane and out-of-plane bending vibrations of the unique conjugated triene structure (–CH=CH–CH_2_–CH=CH–CH=CH–) within the long side chains of urushiol. These characteristic absorption peaks serve as preliminary evidence confirming that urushiol, rosin amine, and paraformaldehyde successfully synthesized the urushiol–rosin amine–benzoxazine (URB) monomer.

The ^1^H NMR spectra of U, RA, and the newly synthesized URB monomer were further characterized using CDCl_3_ as the solvent, as depicted in Figure 2b. In comparison with the ^1^H NMR spectra of U and RA, two distinct new peaks were observed in the ^1^H NMR spectrum of URB, with chemical shifts at 4.81 and 3.90 ppm. These peaks are indicative of the protons in the Ar–CH_2_–N and O–CH_2_–N groups of the oxazine ring within the benzoxazine framework. The integration of these two peaks yielded an area ratio of approximately 1:1. The relative intensity of the absorption peaks and the integration area in the ^1^H NMR spectrum typically reflect the relative abundance of the protons. Given that the number of protons in the Ar–CH_2_–N and O–CH_2_–N groups is in a 1:1 ratio, the integration area ratio of the corresponding absorption peaks is also 1:1. These findings further substantiate the successful synthesis of the URB monomer.

### 2.2. Curing Process of URB Monomers

The newly synthesized URB monomer undergoes thermal ring-opening polymerization (ROP) to yield the corresponding benzoxazine polymer, URHP. The thermal ROP behavior of the URB monomer is investigated using ATR-FTIR, TGA, and DSC. To examine the structural changes in the benzoxazine framework following thermal ROP, ATR-FTIR spectroscopy was employed, with the results presented in Figure 2a. The characteristic absorption peak of the URB monomer at 3012.6 cm^−1^ (corresponding to the isolated unsaturated =C–H group) significantly diminishes. Furthermore, in the ATR-FTIR spectrum of URHP, the characteristic absorption peaks at 981.5 cm^−1^ and 944.9 cm^−1^ (associated with the triene structure in the long side chains of urushiol) vanish. Additionally, the characteristic absorption band of the oxazine ring at 965.2 cm^−1^ and the C–O–C asymmetric stretching vibration peak at 1256.9 cm^−1^, which were present in the URB spectrum, are also absent in the ATR-FTIR spectrum of URHP. Notably, after thermal curing, the intensity of the characteristic absorption peak in the ATR-FTIR spectrum of URHP between 3300 cm^−1^ and 3400 cm^−1^ is markedly enhanced compared to that of URB, attributable to the formation of more free phenolic hydroxyl groups (–OH) through hydrogen bonding following the ROP of the URB monomer. These findings confirm that the oxazine ring within the benzoxazine structure undergoes ROP, resulting in the formation of a cross-linked benzoxazine polymer. Furthermore, the surface chemical composition of URHP coating has been investigated based on EDS and XPS analysis, which presented in Supplementary Materials Figure S2.

**Figure 3:**
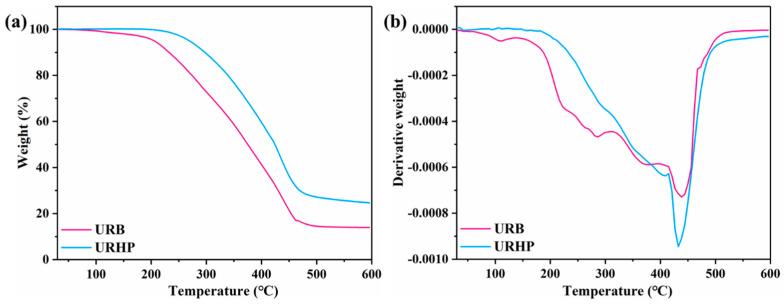
TGA (**a**) and DTG (**b**) curves of UR and URHP.

TGA profiles were recorded to examine the thermal stability, based on the initial decomposition temperature (T_5%_, defined as the temperature corresponding to 5 wt% decomposition) and char yield, of the benzoxazine before and after thermal curing polymerization. The results are presented in Figure 3 and Table 1. It is observed that the initial decomposition temperature of the uncured URB monomer is 205.9 °C, and at the conclusion of the test, the residue carbon content is 13.9 wt%. Following thermal curing ROP, the thermal stability of the resulting URHP shows a notable improvement compared to the uncured sample. The T_5%_ of URHP increases to 269.5 °C, and the residue carbon content rises to 24.7 wt%. Clearly, the complete ROP of the benzoxazine monomer leads to the formation of a cross-linked structure, and the reaction sites on the side chains further crosslink, resulting in a more stable configuration. Additionally, the URB structure exhibits a distinct rigidity, which contributes to the excellent thermal stability and high residue carbon content of the URHP formed after thermal ROP.

**Figure 4:**
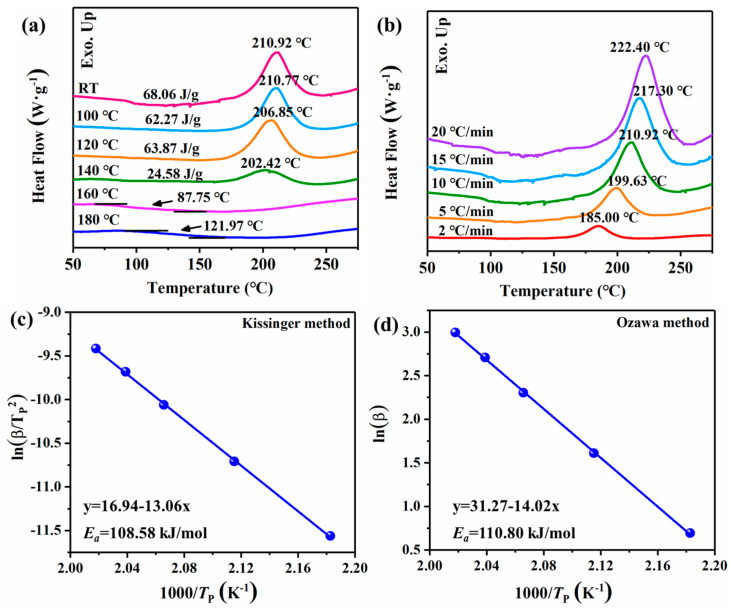
(**a**) DSC thermogram of URB after different temperature treatment. (**b**) Kinetic thermal curing behavior of URB at various heating rates through DSC measurements. Representations of the Kissinger (**c**) and Ozawa (**d**) method for the calculation of the activation energy (*E_a_*) of URB.

DSC testing was employed to characterize the thermal effects of the URB monomer during the thermal ROP process, as shown in Figure 4a. The DSC thermogram revealed a distinct thermal event: a prominent exothermic curing peak observed during the curing of URB. This exothermic peak, occurring at 210.92 °C, can primarily be attributed to the opening of the oxazine ring in the benzoxazine monomers and the formation of the corresponding polymeric product, URHP. Upon heat treatment, the maximum exothermic peak of URB gradually diminishes, suggesting that the structure of URB undergoes alteration following thermal treatment, resulting in a reduction in molecular rigidity and facilitating segmental motion. These structural changes and enhanced segmental mobility influence the ROP process. After heat treatments at 120 °C and 140 °C, the maximum exothermic peak continues to decrease, shifting to 206.85 °C and 202.42 °C, respectively. The progressive decline in the maximum exothermic peak suggests a potential thermal catalytic effect, likely induced by the catalytic action of the phenolic hydroxyl group in the phenolic segment of the URB structure. At heat treatment temperatures of 160 °C and 180 °C, the maximum exothermic peak in the DSC curve vanishes completely, accompanied by the emergence of a higher glass transition temperature (T_g_), indicating the completion of the thermal ROP process and the formation of URHP with enhanced thermal properties.

The activation energy (*E_a_*) for the ROP of newly designed benzoxazine monomers was also investigated with non-isothermal DSC at different heating rates of 2, 5, 10, 15, and 20 °C/min. The DSC thermograms of benzoxazine monomers at different heating rates are shown in Figure 4b. Then, the value of *E_a_* for the ROP process was determined via the well-known Kissinger and Ozawa methods. Based on the Kissinger and Ozawa methods, the value of *E_a_* can be calculated by the following equations:

(1) $$ln\left(\frac{\beta }{T_{P}^{2}}\right)=ln\left(\frac{AR}{E_{a}}\right)-\frac{E_{a}}{RT_{P}}\;\;\;\;\;\;\;\;\;\;\;\mathrm{Kissinger}\;\mathrm{equation}$$

(2) $$\ln\beta =-1.052\frac{E_{a}}{{RT}_{P}}+C\;\;\;\;\;\;\;\;\;\;\;\mathrm{modified}\;\mathrm{Ozawa}\;\mathrm{equation}$$

where *β* represents the heating rate (K·min^−1^), *T_P_* is the temperature at the peak of heat flow (K), *E_a_* is the activation energy (kJ·mol^−1^), *A* is the pre-exponential factor, *R* is the gas constant, and *C* is constant.

At a heating rate of 20 °C/min, the maximum exothermic temperature for the ROP process of URB is observed to be 222.40 °C. For heating rates of 15, 10, 5, and 2 °C/min, the corresponding maximum exothermic temperatures of URB are 217.30, 210.92, 199.63, and 185.00 °C, respectively. As illustrated in Figure 4c,d, linear regression of the heating rates and maximum exothermic temperatures of the URB monomer was conducted according to both the Kissinger and Ozawa theories. The *E_a_* values for URB were determined to be 108.58 and 110.80 kJ/mol, calculated from the slopes of the linear regression obtained using both the Kissinger and Ozawa methods. Notably, previous studies have reported that the *E_a_* value for benzoxazine typically exceeds 150 kJ/mol [32]. These findings suggest that the URB monomer can undergo ROP at a lower temperature, thereby consuming less energy, which can be attributed to the catalytic influence of the potential catalytic group (–OH) present in the urushiol group of the URB monomer. The relevant literature [33,34] indicates that the ROP process of benzoxazine generally follows a self-catalyzed polymerization mechanism, albeit requiring high temperatures (>200 °C). Figure 4 proposes a possible mechanism for the structural transformation of the URB monomer during the ROP process.

**Figure 5:**
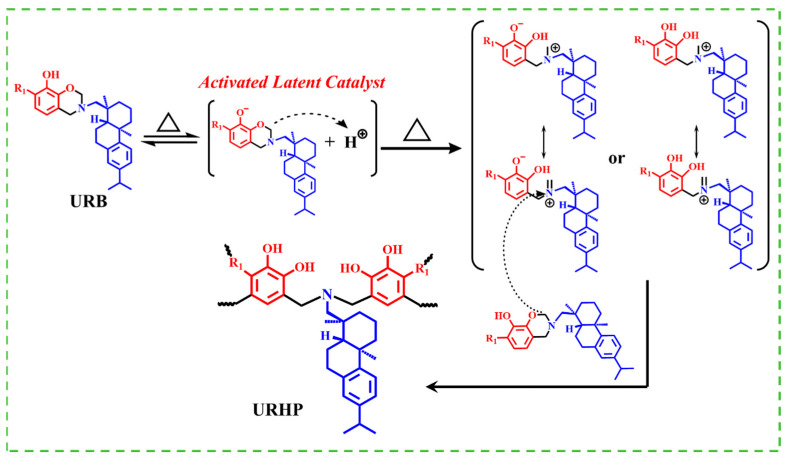
Suggested mechanism of the transformation of URB after ROP.

Based on the DSC and FTIR experimental results, the potential catalytic effect in URB arises from the phenolic hydroxyl group embedded in its structure. As shown in Figure 5, upon thermal treatment, a portion of the URB monomers becomes protonated by hydrogen ions generated from the ionization of other URB monomers. The protonated URB monomers exhibit enhanced reactivity, thereby leading to lower ROP temperatures. This results in the cleavage of the O–CH_2_–N bond in the oxazine ring, giving rise to a cationic imine component of the zwitterionic intermediate. Subsequently, adjacent benzoxazine monomers attack the cationic imine group through electrophilic substitution, inducing a structural rearrangement and the formation of a novel Mannich bridge. Ultimately, the oxazine ring in benzoxazine undergoes continuous ROP under prolonged heating, yielding a crosslinked polymer. Simultaneously, the unsaturated bonds in the urushiol side chains also engage in the reaction, further crosslinking the polymer and rendering URHP a highly crosslinked network structure.

### 2.3. Surface Analysis of URHP Coatings

**Figure 6:**
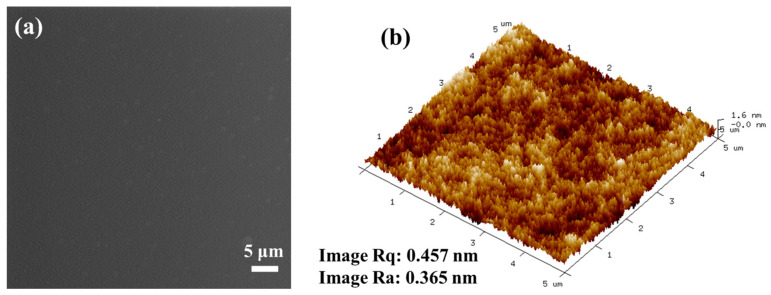
(**a**) FE-SEM image of the surface of URHP coating; (**b**) AFM image of the surface of URHP coating.

The surface of the URHP polymer coating, synthesized via the high-temperature ROP of the URB monomer, was analyzed. Initially, optical images (see Figure S1a) reveal that the surface of URHP coating is smooth, flat, and exhibits high gloss and brightness. Additionally, FE-SEM and AFM were employed to examine the surface morphology of the URHP coating. The FE-SEM images (Figure 6a) present a microscopic morphology that aligns with the optical images, demonstrating a surface that is exceptionally smooth and flat, devoid of fine pores or cracks at various magnifications (×5000, ×10,000, and ×50,000). The AFM images (Figure 6b) further corroborate that the URHP coating maintains a smooth and flat microstructure. The 3D images reveal that the root mean square roughness (Image Rq) of the coating’s micro-surface is 0.457 nm, and the average roughness (Image Ra) is 0.365 nm. These findings reinforce that the URHP coating’s surface is remarkably smooth and flat. According to the literature [35], the smooth surface of the URHP coating is comparable to that of PDMS, which has a root mean square roughness of 0.78 nm. Both the FE-SEM and AFM images confirm that the coating exhibits excellent sealing properties, with no pinholes or cracks observed. These attributes suggest that URHP could serve as a unique impermeable coating, primarily owing to the crosslinking reactions of phenolic hydroxyl groups and the multiple double bonds present in the urushiol structure.

**Figure 7:**
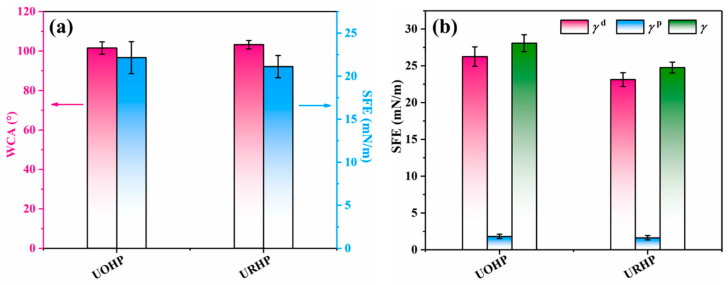
(**a**) The WCA and SFE of UOHP and URH coatings. (**b**) The polar component (*γ*_s_^p^), dispersive component (*γ*_s_^d^), and total surface energy (*γ*_l_) of UOHP and URHP coatings.

The surface free energy of the coating is a crucial factor influencing its wettability and adhesion performance. The literature suggests that lower surface energy leads to reduced fouling adhesion, thereby enhancing antifouling properties [36,37]. Hence, the surface energy of the URHP polymer coating was evaluated by measuring the contact angle of liquids on the solid surface and applying model formulas. To more comprehensively assess the surface free energy of the URHP polymer coating, we selected another urushiol-based benzoxazine polymer (UOHP) previously reported by our research as a reference [28,29]. UOHP is a benzoxazine synthesized from urushiol, octylamine, and paraformaldehyde, and it also employs a thermal ring-opening polymerization method to prepare the polymer coating. Initially, the contact angle of DI water on the coating surface was measured to assess the wettability of the coating, and the surface free energy was calculated using the EOS model, as illustrated in Figure 7a.

The WCA of the URHP coating is 103.2 ± 2.2°, and its contact angle exceeds 98°, rendering the URHP coating a hydrophobic surface. The calculated surface free energy (SFE) of URHP is 21.11 ± 1.3 mN/m, signifying that the URHP coating possesses relatively low surface energy. Additionally, by utilizing diiodomethane as the testing medium, the Owens–Wendt–Kaelble (OWK) model was employed to compute the polar component (*γ*_s_^p^), dispersive component (*γ*_s_^d^), and total surface energy (*γ*) of the URHP coating surface (Figure 7b). These results further corroborate that the URHP coating exhibits relatively low surface energy. Both tests, employing two media and two calculation models, demonstrate that the surface energy of the URHP coating is lower than that of the UOHP coating. A lower surface energy suggests diminished fouling adhesion, thus indicating enhanced antifouling performance potential.

### 2.4. Fouling-Resistant Assays of URHP Coating

**Figure 8:**
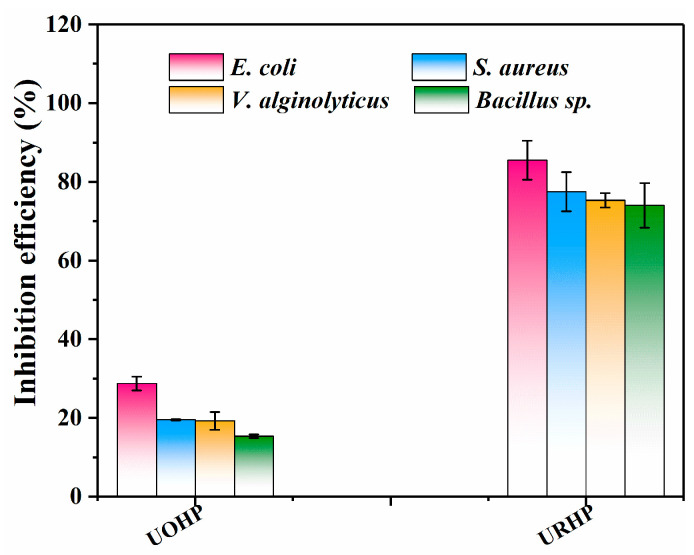
The inhibition efficiency of UOHP and URHP coatings relative to BG towards *E. coli* and *S. aureus*, *V. alginolyticus*, and *Bacillus* sp.

Rosin amine is widely utilized as a disinfectant and algaecide in wastewater treatment. In this section, rosin amine is introduced as the amine source into the structure of benzoxazine to bestow certain antibacterial properties upon the benzoxazine polymer. To investigate the antibacterial efficacy of the URHP coating, typical Gram-negative bacterium *E. coli* (BW25113), Gram-positive bacterium *S. aureus* (ATCC 25923), marine bacteria *V. alginolyticus* (ATCC 33787), and *Bacillus* sp. (MCCC 1B00342) were selected. The antibacterial rate is calculated by counting the number of bacterial colonies grown from bacterial cultures obtained from the surface of the benzoxazine polymer coating samples on agar plates and comparing these to the colonies grown from the control glass slide (BG) sample. The results are presented in Figure 8 and Figure S3. To further elucidate the antibacterial performance of URHP, the previously reported UOHP coating is also used as a control. As depicted in the figure, bacterial cultures separated from the BG sample yield a large number of *E. coli*, *S. aureus*, *V. alginolyticus*, and *Bacillus* sp. colonies on the agar plate; the colony counts are (5.75 ± 0.85) × 10^5^ CFU/mL, (7.31 ± 1.78) × 10^5^ CFU/mL, (9.58 ± 2.36) × 10^5^ CFU/mL, and (6.24 ± 1.59) × 10^5^ CFU/mL, respectively, indicating that the BG sample lacks bactericidal properties. Similarly, the UOHP coating also supports the growth of a large number of bacterial colonies on the agar plate; the colony counts of *E. coli*, *S. aureus*, *V. alginolyticus*, and *Bacillus* sp. are (4.09 ± 1.35) × 10^5^ CFU/mL, (5.88 ± 2.78) × 10^5^ CFU/mL, (7.74 ± 3.36) × 10^5^ CFU/mL, and (5.28 ± 1.95) × 10^5^ CFU/mL, respectively. However, the colony counts of *E. coli*, *S. aureus*, *V. alginolyticus*, and *Bacillus* sp. isolated from URHP coatings are (0.83 ± 1.15) × 10^5^ CFU/mL, (1.64 ± 0.98) × 10^5^ CFU/mL, (2.37 ± 1.45) × 10^5^ CFU/mL, and (1.62 ± 0.95) × 10^5^ CFU/mL, respectively. In comparison to the BG sample, the antibacterial rates of the UOHP coating against *E. coli*, *S. aureus*, *V. alginolyticus*, and *Bacillus* sp. are 28.75 ± 1.77%, 19.50 ± 0.14%, 19.24 ± 2.24%, and 15.35 ± 0.49%, respectively, suggesting that the antibacterial performance of the UOHP coating is relatively weak. Conversely, for the URHP coating, the number of bacterial colonies on the agar plate is significantly reduced. In comparison to the BG sample, the antibacterial rates of the URHP coating against *E. coli*, *S. aureus*, *V. alginolyticus*, and *Bacillus* sp. are 85.50 ± 4.95%, 77.50 ± 4.95%, 75.30 ± 1.84%, and 74.00 ± 5.66%, respectively, demonstrating that URHP exhibits excellent antibacterial performance. The presence of the rosin amine structure significantly enhances the antibacterial performance compared to the UOHP coating.

The rosin amine utilized in this study is specifically dehydro-rosin amine, a diterpene amine with a tricyclic phenanthrene structure, synthesized through the dehydrogenation and amination of natural rosin acid. This modified rosin product is the principal component of disproportionated rosin. Commonly employed as a wood preservative, as well as a bactericidal and algaecidal agent in industrial wastewater treatment, dehydro-rosin amine and its derivatives also exhibit certain biological activities, including anti-inflammatory and anti-tumor effects. By incorporating the RA structure into the benzoxazine framework, it becomes part of the benzoxazine polymer coating. Upon contact with bacterial cells, the RA structure interferes with the bacteria’s environment and normal metabolic functions, leading to metabolic disruption and preventing the proper conversion of genetic material and energy, ultimately resulting in bacterial cell death. However, due to the limited quantity of rosin amine incorporated into the polymer coating, the antibacterial rate does not exceed 99%. Future research will focus on further enhancing the antibacterial performance of the benzoxazine polymer coating.

**Figure 9:**
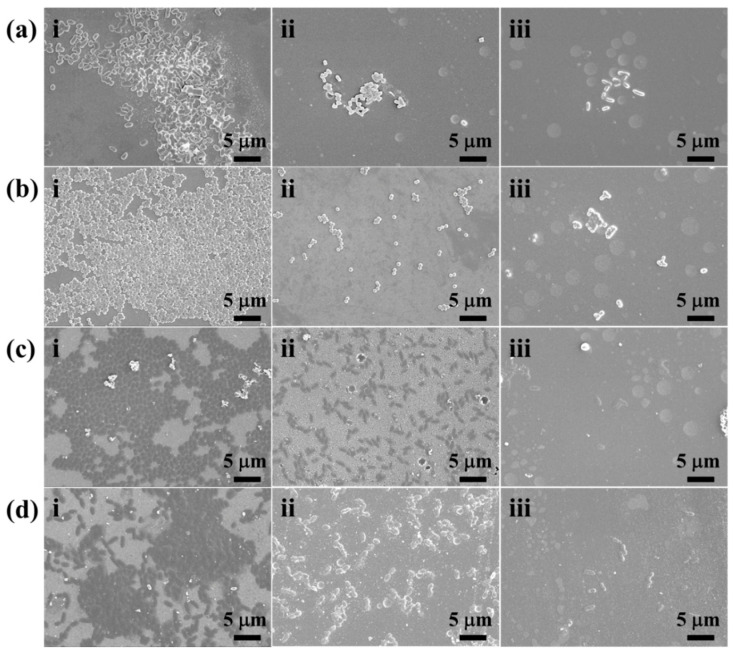
Adhesion of bacteria (**a**) *E. coli*, (**b**) *S. aureus*, (**c**) marine bacterial *V. alginolyticus*, and (**d**) *Bacillus* sp. after 24 h of incubation period on surface of (**i**) BG, (**ii**) UOHP, and (**iii**) URHP coatings by FE-SEM images (the scale bars are 5 μm).

To further assess the antibacterial efficacy of the coating, SEM was employed to observe the adhesion of various bacteria on the coating surface, as depicted in Figure 9. Besides, an additional set of SEM images was included in the Supplementary Information (see Figures S4–S7) The results reveal that all four bacterial types exhibit significant adhesion to both the BG and UOHP surfaces. However, the physicochemical characteristics of the BG and UOHP surfaces differ considerably. The surface energy of UOHP is lower than that of BG, which results in markedly reduced bacterial adhesion on the UOHP surface in comparison to the BG surface. Conversely, bacterial adhesion on the URHP surface is even less than that on the UOHP surface. This is principally attributed to the pronounced antibacterial effect of the URHP coating, which inhibits bacterial growth and, coupled with the low surface energy, leads to further reduction in bacterial adhesion, thereby demonstrating excellent antibacterial and antifouling properties.

**Figure 10:**
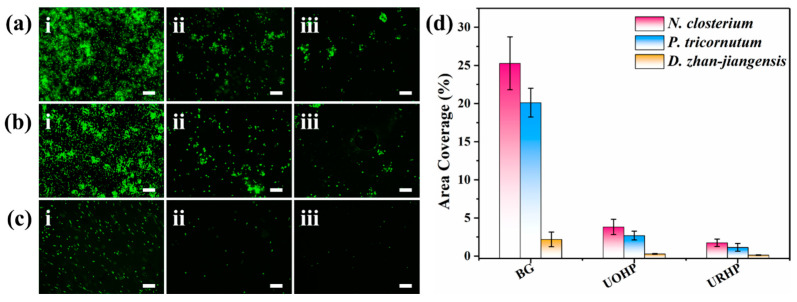
Fluorescent photographs of (**a**) *N. closterium*, (**b**) *P. tricornutum*, and (**c**) *D. zhan-jiangensis* adhesion after 7 days of cultivation time on (**i**) BG, (**ii**) UOHP, and (**iii**) URHP coatings (the scale bars are 100 μm); (**d**) statistical chart showing algal density in examined fields by ImageJ 1.52a software.

**Figure 11:**
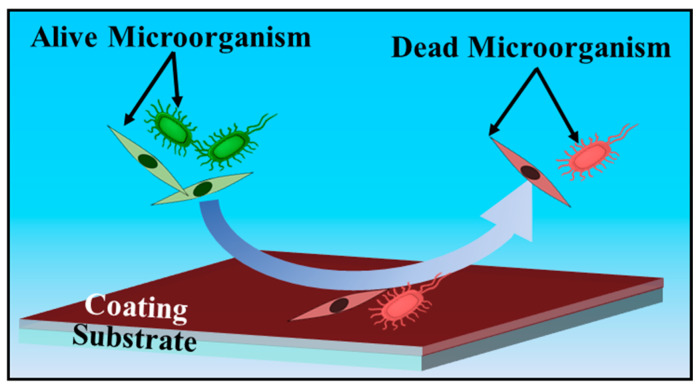
Antifouling mechanism of URHP coating.

Microalgae represent an undesirable form of colonization on artificial surfaces in marine environments, as they readily settle on a variety of surfaces once the marine conditioning biofilm has formed. Consequently, microalgae were selected as a model fouling species to further investigate the biofouling resistance of the newly designed benzoxazine polymer coating. The URHP coating, along with control samples (BG and UOHP), was separately immersed in three marine microalgae species (*N. closterium*, *P. tricornutum*, and *D. zhan-jiangensis*) for co-cultivation over a specified period. The adhesion behavior of the microalgae cells on the coating surface was observed. A fluorescence microscope was utilized to capture the microalgae adhesion on the sample surface, and ImageJ 1.52a software was employed to analyze the fluorescence intensity, quantifying the coverage of the microalgae cells on the coating surface, as shown in Figure 10. In comparison to the BG sample, the microalgae cell adhesion on the UOHP and URHP surfaces was significantly reduced. The coverage of the three algae species on the BG sample surface was 25.3 ± 3.5% (*N. closterium*), 20.1 ± 1.9% (*P. tricornutum*), and 2.2 ± 1.0% (*D. zhan-jiangensis*). On the UOHP surface, the coverage was 3.8 ± 1.0% ((*N. closterium*), 2.7 ± 0.6% (*P. tricornutum*), and 0.3 ± 0.05% (*D. zhan-jiangensis*). On the URHP surface, it was 1.7 ± 0.5% (*N. closterium*), 1.1 ± 0.5% (*P. tricornutum*), and 0.1 ± 0.04% (*D. zhan-jiangensis*). These results are primarily attributed to the low surface energy of the coating, which weakens the adhesion of microalgae cells, facilitating their detachment and demonstrating excellent detachment performance. Furthermore, because the URHP coating possesses inherent antibacterial properties, it also affects the activity of the microalgae cells, resulting in further reduced adhesion on the URHP surface, thereby showcasing superior antifouling performance. Based on these experimental findings, Figure 11 presents a proposed antifouling mechanism for the URHP coating. In the absence of the release of antifouling agents, the intrinsic antimicrobial properties of the URHP coating can effectively kill or repel fouling organisms, while its low surface energy prevents the settlement and adhesion of fouling organisms. Consequently, the synergistic effect of both properties enhances the antifouling performance of the coating.

## 3. Materials and Methods

### 3.1. Materials

Urushiol (U) was extracted from raw lacquer (purchased from Maoba Town, Hubei Province, China) using ethanol as described in the literature [28,38]. The chemical structure of urushiol is shown in Figure 1a. Analytical grade paraformaldehyde, 1,4-dioxane, dichloromethane, diiodomethane, xylene, ethanol, and anhydrous sodium sulfate were purchased from Sinopharm Chemical Reagent Co., Ltd. (Shanghai, China). Rosin amine was purchased from Shanghai Haohong Biomedical Technology Co., Ltd. (Shanghai, China). Unless otherwise specified, all chemicals were used as received without further purification. Deionized (DI) water was prepared in the laboratory using a Millipore Direct-Q3 UV ultrapure water system (Merck, Kennyworth, NJ, USA). Phosphate-buffered saline (PBS, pH = 7.4) was purchased from Shanghai Sangon Biotech Co., Ltd. (Shanghai, China). Artificial seawater (ASW) was prepared according to ASTM D1141-1998 (2013) [39]. *Escherichia coli* (*E. coli*), *Staphylococcus aureus* (*S. aureus*), and *Vibrio alginolyticus* (*V. alginolyticus*) were purchased from BeNa Chuanglian Biotechnology Co., Ltd. (Shangcheng, China). *Bacillus* sp. was purchased from Marine Culture Collection of China (Xiamen, China). *Nitzschia closterium* (*N. closterium*), *Phaeodactylum tricornutum* (*P. tricornutum*), and *Dicrateria zhanjiangensis* (*D. zhan-jiangensis*) were purchased from the Seaweed Culture Collection Center, Institute of Oceanology, Chinese Academy of Sciences (Qingdao, China).

### 3.2. Synthesis of Urushiol–Rosin Amine–Benzoxazine Monomer (URB)

Urushiol–rosin amine–benzoxazine monomer (URB) was prepared using a previously established procedure [28,38]. Briefly, paraformaldehyde (0.15 mol, 4.50 g) was added to a 250 mL three neck round bottom flask equipped with a thermometer, a reflux condenser, and a dropping funnel. Then, rosin amine (0.05 mol, 14.27 g) and 1,4-dioxane (20 mL) were added to the flask, and the mixture was stirred at room temperature for 40 min. Urushiol (0.05 mol, 15.70 g) was dissolved in 1,4-dioxane (20 mL) and added dropwise to the reaction system within 20 min. The reaction system was subsequently gradually heated to 90 °C and reacted for 5 h under vigorous stirring. Afterwards, the reaction mixture was cooled down to room temperature, and the solvent was removed by vacuum distillation. The residual mixture was dissolved in dichloromethane (100 mL), and the solution was washed three times with deionized (DI) water, followed by drying with anhydrous sodium sulfate overnight. Finally, the solvent was removed by rotary evaporation, and a reddish-brown viscous product was obtained, which was dried under vacuum at room temperature for 24 h. The yield of the product is 87% and was named URB.

### 3.3. Urushiol–Rosin Amine–Benzoxazine Polymer (URHP) Coatings

The curing reaction for URHP coatings were prepared by a thermosetting ring-opening polymerization method with the following procedure: a certain amount of URB monomer and xylene were added to a beaker and stirred until a homogeneous solution (40 wt.%) was obtained. The solution was then cast onto glass slides (for surface wettability, bacteria-resistant assay, and algal inhibition assay). Bare glass slides (BG, 2.5 cm × 2.5 cm) were sequentially ultrasonically cleaned with acetone, anhydrous ethanol, and DI water for 10 min and then dried under N_2_ flow to remove any surface contaminants. Nitrogen gas was supplied by Fuzhou Yuanhua Chemical Co., Ltd. (Fuzhou, China), with a purity of 99.99%. The resulting coating was first pre-cured at room temperature for 1 h to allow for complete evaporation of the solvent, which was then subjected to stepwise curing at 100 °C, 120 °C, 140 °C, 160 °C, and 180 °C for 1 h each in an oven until fully cured. The sample was then allowed to cool slowly to room temperature, resulting in a dark brown polymer coating (see Figure 6a). The average thickness of the coating was approximately 50 μm, measured by a Qnix^®^ 4500 coating thickness gauge (Qnix, Cologne, Germany) according to ASTM B499-2009 (2014) [40].

### 3.4. Characterizations

ATR-FTIR spectra were recorded on a Nicolet iS50 FTIR Spectrometer (Thermo Fisher, Waltham, MA, USA) in the range of 400–4000 cm^−1^, resolution of 4 cm^−1^ and 32 scans. ^1^H NMR spectra were recorded on a Bruker 400 MHz NMR spectrometer (Bruker, Billerica, MA, USA), with CDCl_3_ as the solvent. The surface morphology of the URHP coating was characterized by a field emission scanning electron microscope (FE-SEM, Phenomenon, Rotterdam, The Netherlands) with an acceleration voltage of 5 kV. Energy-Dispersive Spectroscopy (EDS) was used to analyze the elemental composition and distribution of the URHP coating surface, with an acceleration voltage of 15 kV, in the FE-SEM (Phenomenon, The Netherlands). The elemental composition and electronic binding energy of the URHP coating surface were characterized by X-ray Photoelectron Spectroscopy (XPS, Escalab Xi+, Thermo Scientific, Waltham, MA, USA), with all binding energies calibrated by the C1s peak at 284.8 eV. The surface morphology and roughness of the URHP coating were characterized by Atomic Force Microscopy (AFM, Bruker Multimode 8, Billerica, MA, USA). The thermal stability of the URB monomer and URHP coating was tested on a METTLER TGA2 thermogravimetric analyzer (Mettler-Toledo, Greifensee, Switzerland) from 30 to 600 °C at a heating rate of 10 °C·min^−1^ under nitrogen atmosphere with a gas flow rate of 50 mL/min, and approximately 5–10 mg of sample. The curing behavior of URB monomer was characterized using a Differential Scanning Calorimeter (DSC) measurement conducted on a DSC2500 (TA, New Castle, DE, USA) from 50 to 280 °C at different heating rates under a nitrogen atmosphere with a gas flow rate of 50 mL/min and approximately 5–10 mg of sample. The surface wettability of the URHP coating was characterized by static water contact angle measurements determined by a drop shape analyzer DSA 25 instrument (Kruss, Hamburg, Germany) using a 2 μL DI water droplet at ambient temperature. Meanwhile, the surface free energy of the coating was calculated using the EOS model. The average value of each sample was calculated by averaging five data points. Further, the surface polarity (γ_s_^p^), dispersion (γ_s_^d^), and total (γ_l_) surface energy of the URHP coating were calculated using the two-liquid Owens–Wendte–Kaelble (OWK) model, with 2 µL of DI water and diiodomethane as the testing media.

### 3.5. Antibacterial Assessments

The antibacterial performance of the URHP coating was evaluated using two typical bacteria, Gram-negative *E. coli* BW25113 and Gram-positive *S. aureus* ATCC 25923, and the marine bacterial species *V. alginolyticus* ATCC 33787 (Gram-negative bacteria) and *Bacillus* sp., MCCC 1B00342 (Gram-positive bacteria), according to the previous procedure [10,41]. The freezing stocks of *E. coli* and *S. aureus* bacterial strains were maintained at −80 °C in 1:1 solution of Luria–Bertani (LB) broth: 40% (*v*/*v*) glycerol. Prior to use in antibacterial tests, the bacterial strains were first cultured in fresh LB broth at 37 °C by shaking at 170 rpm for 20 h, until the O.D._600nm_ of 1.8–2.0 was reached. The O.D._600nm_ was measured using an ultraviolet-visible spectrophotometer (UV-2600i, Shimadzu, Kyoto, Japan). The *V. alginolyticus* and *Bacillus* sp. was stored frozen at −80 °C in a solution of 2216E medium and glycerol; similarly, *V. alginolyticus* preactivated in fresh 2216E medium at 30 °C and *Bacillus* sp. at 28 °C. Then, the BG and URHP coating (7.5 cm × 2.5 cm) were cleaned by wiping with anhydrous ethyl alcohol, sterilized with ultraviolet radiation for 30 min, and placed in plastic petri dishes. The suspensions of bacteria were diluted to ca. 10^5^−10^6^ CFU/mL in fresh medium, determined using a hemocytometer (Shanghai Qiujing Biochemical Reagent Instrument Co., Ltd., Shanghai, China). Subsequently, the BG and URHP coating were inoculated with 500 μL of diluted bacterial suspension and covered with plastic wraps. The bacterial strains were incubated at 37 °C (*E. coli* and *S. aureus*), 30 °C (*V. alginolyticus*), and 28 °C (*Bacillus* sp.) for 24 h under static condition. Afterwards, the plastic wraps were removed, and the plates were gently rinsed with 20 mL of sterile phosphate-buffered saline (PBS) to ensure that non-adhered bacteria were washed away. The washed bacteria were cultured on agar plates, and the number of colonies was counted. The antibacterial rate (A.R.) of the URHP coating was calculated by counting the colonies present on the agar plate from the PBS wash, using the following equation [42,43]:

(3) $$A.R.=\frac{N_{control}-N_{sample}}{N_{control}}\times 100\%$$

where A.R. represents the antibacterial rate (%) of the URHP coating, *N_control_* is the colony number (CFU/mL) of the BG sample on the agar plate, and *N_sample_* is the colony number (CFU/mL) of the URHP sample on the agar plate. Each group of samples was tested three times to obtain the average value and standard deviation.

To further observe bacterial adhesion on the sample surfaces, the PBS-washed BG and URHP coating were fixed with 2.5% glutaraldehyde, followed by gradient dehydration with ethanol solutions of varying concentrations. After freeze-drying, the sample surfaces were gold-coated and bacterial adhesion on the BG and URHP surfaces was observed by FE-SEM (SU8010, Hitachi, Tokyo, Japan).

### 3.6. Algal Biofouling Assessments

To evaluate the effects of the URHP coating on algae, algal growth and attachment experiments were conducted. Algal cells of *N. closterium*, *P. tricornutum*, and *D. zhan-jiangensis* cells were grown in f/2 culture media, which were prepared in ASW at 22 ± 2 °C under a cycle of 12 h of fluorescent light and 12 h of dark on a biological incubator. After 7 days of growth, the culture media containing algal cells were diluted with fresh culture media to give the test media with the concentrations of algal cells at 10^5^−10^6^/mL, which were used for the following algal attachment experiments.

Similar to the antibacterial assessments, the sterilized BG and the URHP coating were immersed in a glass petri dish containing 30 mL of culture media inoculated with *N. closterium*, *P. tricornutum*, and *D. zhan-jiangensis* cells. After being immersed for 7 days and settling down, the BG and the URHP coating were taken out from the test media and rinsed with 20 mL of sterile PBS to wash away any non-adhered algae. Subsequently, the algae adhered onto the surfaces of the BG and the URHP coating were examined using a fluorescence microscope (Eclipse Ci-L plus, Nikon, Tokyo, Japan), and the images of five random fields (40× magnification, 0.156 mm^2^/per field) were recorded for each sample. The algal coverage over BG and the URHP coating was determined by analyzing the fluorescence microscope images using the ImageJ 1.52a software. The experiment was performed in triplicate to obtain the standard deviation.

## 4. Conclusions

By utilizing rosin amine, which possesses inherent antibacterial properties, as the amine source, the urushiol–rosin amine–benzoxazine monomer was successfully synthesized. This monomer was subsequently subjected to thermal ring-opening polymerization (ROP) to yield urushiol–rosin amine–benzoxazine polymer coatings (URHP). Due to the potential catalytic effect of the phenolic hydroxyl group in urushiol, the ROP temperature of the URB was relatively low, and its activation energy, 108.58 kJ/mol using the Kissinger method and 110.80 kJ/mol using the Ozawa method, was slightly lower than those reported in the literature. The URHP coating exhibited a smooth and uniform surface that is impermeable. The contact angle of URHP was 103.2 ± 2.2°, and the surface energy was 21.11 ± 1.3 mN/m, which revealed that the URHP coating is hydrophobic with low surface energy, making it particularly suitable for antifouling applications. Furthermore, the URHP coating demonstrated excellent anti-adhesion properties against four types of bacteria and three species of marine microalgae. Simultaneously, the URHP coating exhibited remarkable antibacterial performance against four distinct bacterial strains, thereby confirming that the incorporation of antimicrobial structures into the benzoxazine framework is a viable strategy for developing intrinsically antimicrobial benzoxazine polymer coatings. Future research will focus on the development of benzoxazine coatings with enhanced intrinsic antibacterial properties, aimed at the continuous improvement of antimicrobial performance. Additionally, the introduction of a benzoxazine-based polymer coatings with self-healing capabilities is anticipated. This non-leaching antibacterial approach not only enhances the static antifouling efficacy of the coatings but also provides a more environmentally friendly and sustainable solution compared to methods that enhance static antifouling through the release of antimicrobial agents. Thus, this strategy presents a green, eco-friendly solution for antifouling applications.

## Figures and Tables

**Table 1:** Thermal-stability parameters of URB and URHP derived from TGA and DTG curves.

Samples	T_5%_ (°C)	T_max_ (°C)	Residue at 600 °C (wt%)
URB	205.9	438.3	13.9
URHP	269.5	432.6	24.7

## Data Availability

Data will be made available on request.

## Supplementary Materials

The following supporting information can be downloaded at: https://www.mdpi.com/article/10.3390/ijms26094118/s1.

## Abbreviations

The following abbreviations are used in this manuscript:

URB	Urushiol-based benzoxazine
URHP	Urushiol-based benzoxazine polymer
U	Urushiol
RA	Rosin amine
DI	Deionized
ASW	Artificial seawater
*E. coli*	*Escherichia coli*
*S. aureus*	*Staphylococcus aureus*
*V. alginolyticus*	*Vibrio alginolyticus*
*N. closterium*	*Nitzschiaclosterium*
*P. tricornutum*	*Phaeodactylumtricornutum*
*D. zhan-jiangensis*	*Dicrateria zhanjiangensis*
LB	Luria-Bertani
PBS	Phosphate-buffered saline
A.R.	Antibacterial rate
BG	Bare glass slide
ROP	Ring-opening polymerization
PDMS	Polydimethylsiloxane
UOHP	Urushiol-based benzoxazine polymer
ATR-FTIR	Attenuated Total Reflection Flourier Transformed Infrared
NMR	Nuclear Magnetic Resonance
FE-SEM	Field emission scanning electron microscope
EDS	Energy-Dispersive Spectroscopy
XPS	X-ray Photoelectron Spectroscopy
AFM	Atomic Force Microscopy
DSC	Differential Scanning Calorimeter
EOS	Equation of State
OWK	Owens–Wendte–Kaelble
TGA	Thermal Gravimetric Analyzer
DTG	Derivative Thermogravimetry
