# Supplementary material for: Intrinsic Antibacterial Urushiol-Based Benzoxazine Polymer Coating for Marine Antifouling Applications
Source: Int J Mol Sci. 2025 Apr 26;26(9):4118. doi: 10.3390/ijms26094118 (PMC12071434; doi:10.3390/ijms26094118)
Supplement: Supplementary file 1 [file ijms-26-04118-s001.zip › ijms-3568904-supplementary.pdf]

Supporting Information

Intrinsic Antibacterial Urushiol-Based Benzoxazine Polymer Coating for Marine Antifouling Applications

Nuo Chen [1,†], Jide Zhu [2,†], Xinrong Chen [1], Fengcai Lin [1], Xiaoxiao Zheng [1], Guocai Zheng [1], Qi Lin [1], Jipeng Chen [1,*] and Yanlian Xu [1,*]

[1] Fujian Engineering Research Center of New Chinese Lacquer Materials, College of Materials and Chemical Engineering, Minjiang University, Fuzhou 350108, China; ccccheng7788@outlook.com (N.C.); 13599930822@163.com (X.C.); fengcailin@mju.edu.cn (F.L.); xxzheng@mju.edu.cn (X.Z.); 2231@mju.edu.cn (G.Z.); qlin1990@163.com (Q.L.)
[2] National Engineering Research Center of Chemical Fertilizer Catalyst (NERC-CFC), College of Chemical Engineering, Fuzhou University, Fuzhou 350002, China; zjd320610217@163.com
* Correspondence: jpchen@mju.edu.cn (J.C.); ylxu@mju.edu.cn (Y.X.)
† These authors contributed equally to this work.

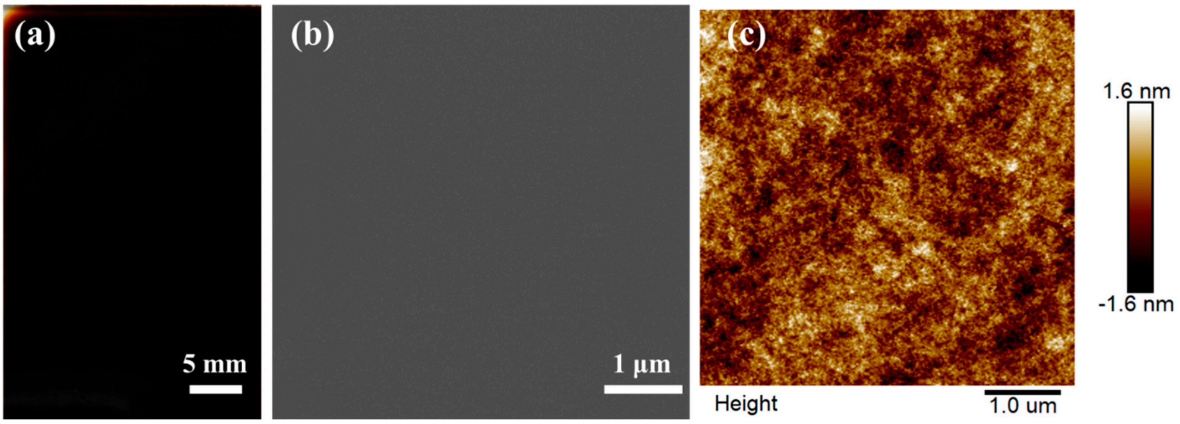

Figure S1. (a) optical image of URHP coating; (b) FE-SEM image of the surface of URHP coating; and (c) AFM image of the surface of URHP coating.

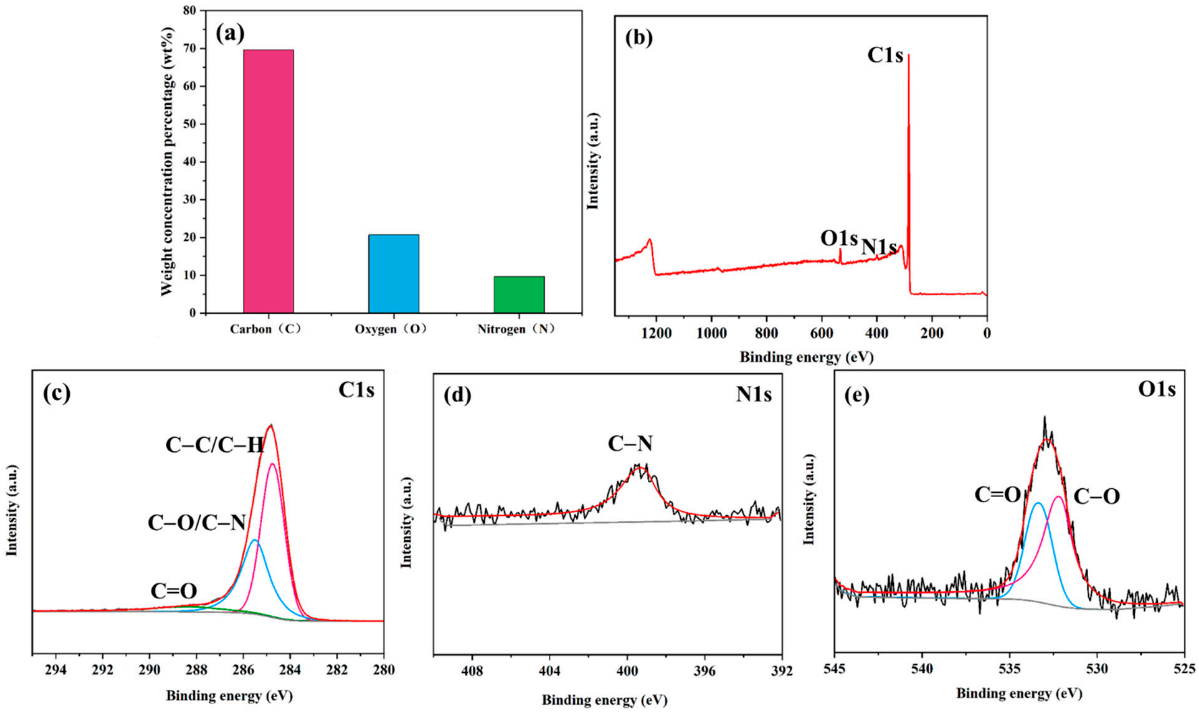

Figure S2. (a) The surface chemical composition of URHP coating based on EDS analysis. (b) XPS survey spectra of URHP coating and XPS high resolution spectra of URHP coating, (c) C1s, N1s and O1s.

EDS and XPS were employed to analyze the elemental composition and electronic binding energy of the URHP coating surface, as shown in **Figure S2**. The EDS mapping images of C, N, and O elements (**Figure S2a**) reveal that all elements are evenly distributed across the measured region of the URHP coating surface, with a predominant presence of C and O elements, at 69.61 wt% and 20.73 wt%, respectively, and a comparatively smaller amount of N element (9.66 wt%), primarily attributed to the low nitrogen content in the URB structure. XPS characterization results further corroborate the elemental composition of the URHP coating surface. The XPS survey spectrum (**Figure S2b**) demonstrates a substantial amount of C and O elements on the surface, with corresponding absorption peaks at 284.9 and 532.4 eV, respectively, and a minor presence of

N element with a peak at 399.7 eV. The peak fitting of the high-resolution XPS spectra for C1s, N1s, and O1s reveals that the chemical environments of C elements include C−C, C−H, C−N, C−O, and C=O, while the fitting peaks for N1s and O1s at 399.3 and 533.3, 532.2 eV correspond to the chemical environments of N and O elements, specifically C−N, C−OH, and C=O, respectively. Therefore, by integrating the ATR-FTIR spectrum results of the URHP coating with the surface elemental composition and chemical environment analysis, it is confirmed that URB undergoes high-temperature ROP to yield URHP, resulting in a smooth, flat, and impermeable URHP coating.

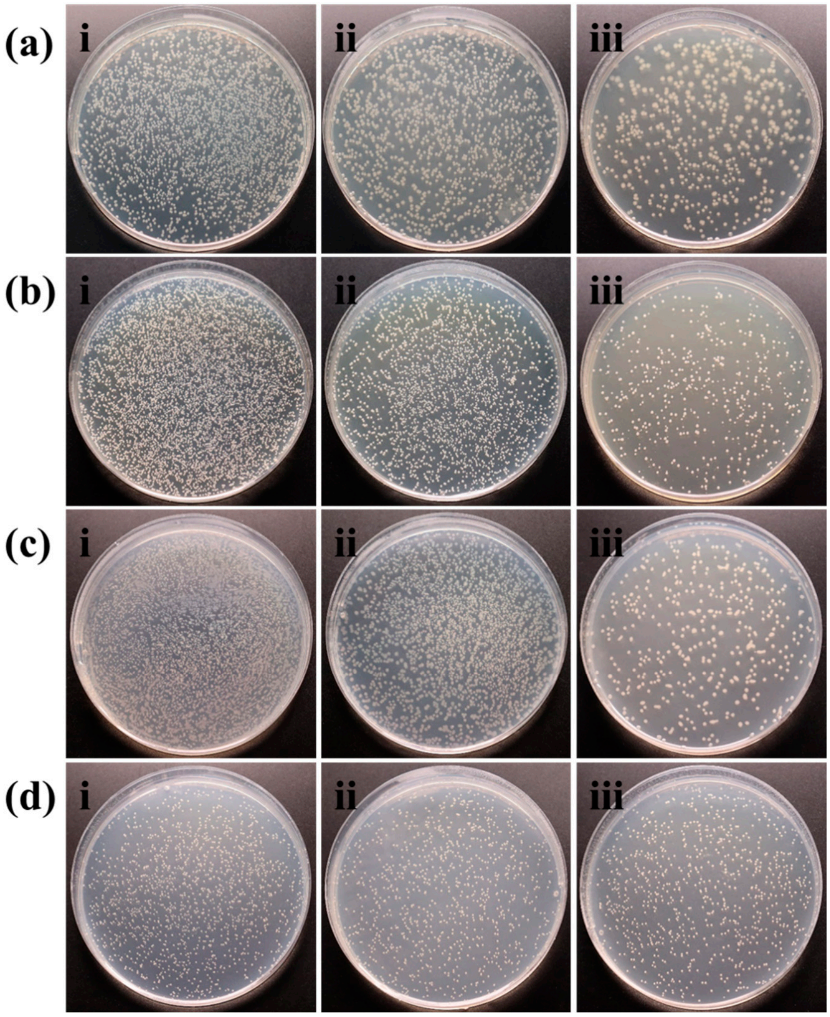

Figure S3. Digital photographs of antibacterial test towards typical (a) Gram-negative bacteria *E. coli*, (b) Gram-positive bacteria *S. aureus*, (c) marine bacterial *V. alginolyticus* and (d) *Bacillus sp.* after 24 h of incubation on (i) BG, (ii) UOHP, (iii) URHP.

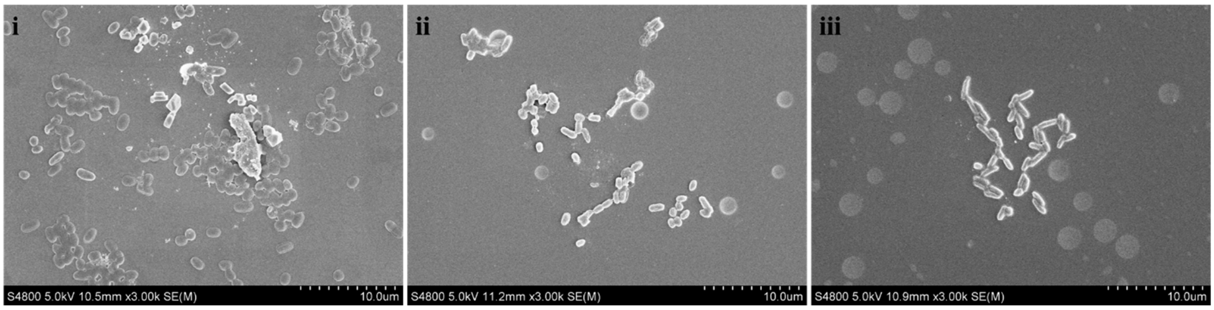

Figure S4. Adhesion of *E. coli* after 24 h of incubation period on surface of (i) BG, (ii) UOHP, (iii) URHP coatings by FE-SEM images.

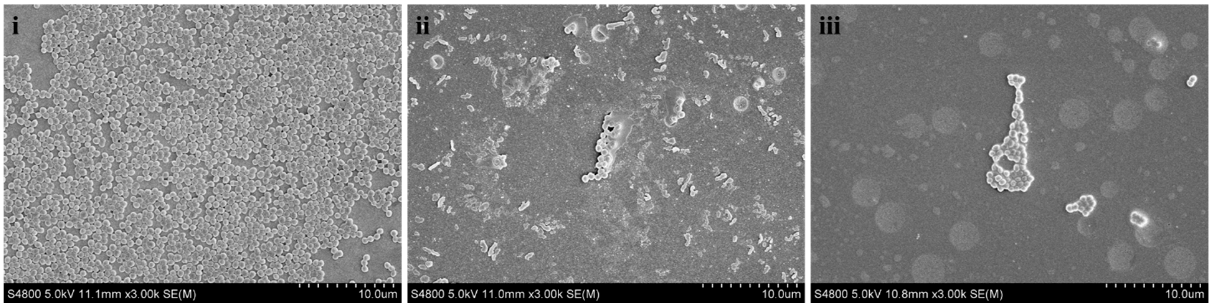

Figure S5. Adhesion of *S. aureus* after 24 h of incubation period on surface of (i) BG, (ii) UOHP, (iii) URHP coatings by FE-SEM images.

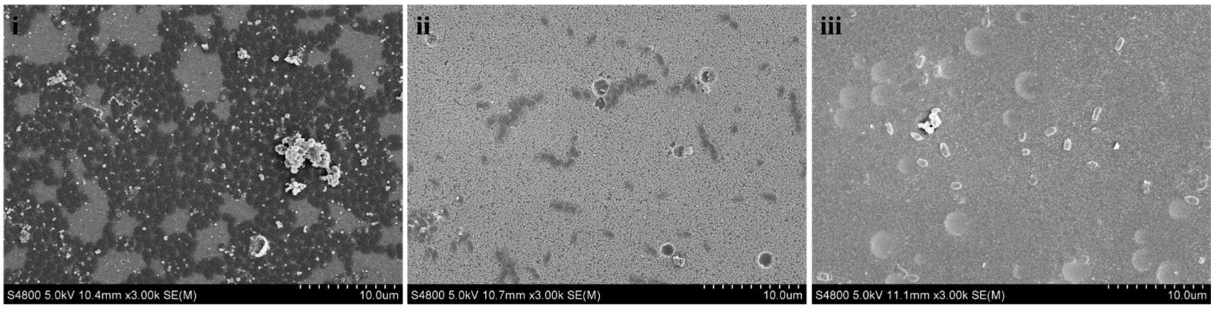

Figure S6. Adhesion of *V. alginolyticus* after 24 h of incubation period on surface of (i) BG, (ii) UOHP, (iii) URHP coatings by FE-SEM images.

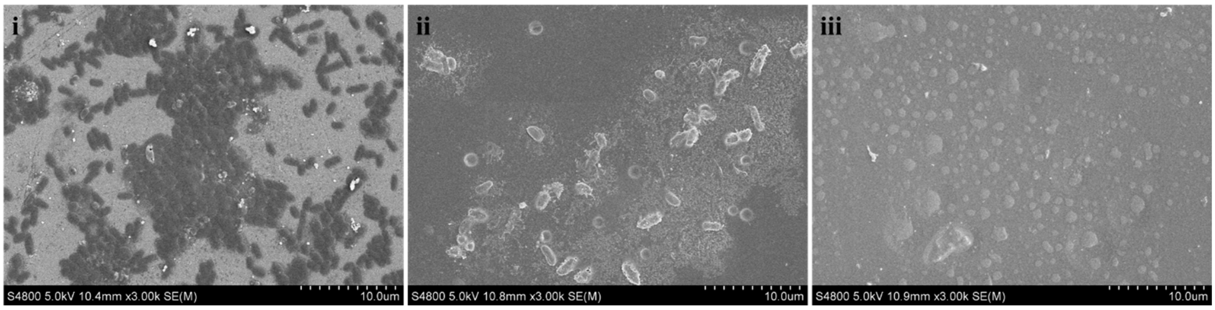

Figure S7. Adhesion of *Bacillus sp.* after 24 h of incubation period on surface of (i) BG, (ii) UOHP, (iii) URHP coatings by FE-SEM images.
